# Extraordinary Titer and Broad Anti-SARS-CoV-2 Neutralization Induced by Stabilized RBD Nanoparticles from Strain BA.5

**DOI:** 10.3390/vaccines12010037

**Published:** 2023-12-28

**Authors:** Zhantong Wang, Baoshan Zhang, Li Ou, Qi Qiu, Lingshu Wang, Tatsiana Bylund, Wing-Pui Kong, Wei Shi, Yaroslav Tsybovsky, Lingyuan Wu, Qiong Zhou, Ridhi Chaudhary, Misook Choe, Thayne H. Dickey, Mohammed El Anbari, Adam S. Olia, Reda Rawi, I-Ting Teng, Danyi Wang, Shuishu Wang, Niraj H. Tolia, Tongqing Zhou, Peter D. Kwong

**Affiliations:** 1 Vaccine Research Center, National Institute of Allergy and Infectious Diseases, National Institutes of Health, Bethesda, MD 20892, USA; zhantong.wang@nih.gov (Z.W.); qi.qiu@nih.gov (Q.Q.); tatsiana.bylund@nih.gov (T.B.); lingyuan.wu@nih.gov (L.W.); misook.choe@nih.gov (M.C.); danyi.wang@nih.gov (D.W.); shuishu.wang@nih.gov (S.W.); 2 Vaccine Research Center Electron Microscopy Unit, Cancer Research Technology Program, Frederick National Laboratory for Cancer Research, Frederick, MD 20701, USA; 3 Host-Pathogen Interactions and Structural Vaccinology Section, Laboratory of Malaria Immunology and Vaccinology, National Institute of Allergy and Infectious Diseases, National Institutes of Health, Bethesda, MD 20892, USA; thayne.dickey@nih.gov (T.H.D.);

**Keywords:** COVID-19, encapsulin, immunization, nanoparticle vaccine, neutralizing response, RBD, SARS-CoV-2, SpyTag:SpyCatcher conjugation

## Abstract

The receptor-binding domain (RBD) of the SARS-CoV-2 spike is a primary target of neutralizing antibodies and a key component of licensed vaccines. Substantial mutations in RBD, however, enable current variants to escape immunogenicity generated by vaccination with the ancestral (WA1) strain. Here, we produce and assess self-assembling nanoparticles displaying RBDs from WA1 and BA.5 strains by using the SpyTag:SpyCatcher system for coupling. We observed both WA1- and BA.5-RBD nanoparticles to degrade substantially after a few days at 37 °C. Incorporation of nine RBD-stabilizing mutations, however, increased yield ~five-fold and stability such that more than 50% of either the WA1- or BA.5-RBD nanoparticle was retained after one week at 37 °C. Murine immunizations revealed that the stabilized RBD-nanoparticles induced ~100-fold higher autologous neutralization titers than the prefusion-stabilized (S2P) spike at a 2 μg dose. Even at a 25-fold lower dose where S2P-induced neutralization titers were below the detection limit, the stabilized BA.5-RBD nanoparticle induced homologous titers of 12,795 ID_50_ and heterologous titers against WA1 of 1767 ID_50_. Assessment against a panel of β-coronavirus variants revealed both the stabilized BA.5-RBD nanoparticle and the stabilized WA1-BA.5-(mosaic)-RBD nanoparticle to elicit much higher neutralization breadth than the stabilized WA1-RBD nanoparticle. The extraordinary titer and high neutralization breadth elicited by stabilized RBD nanoparticles from strain BA.5 make them strong candidates for next-generation COVID-19 vaccines.

## 1. Introduction

While vaccines have been instrumental in ending the COVID-19 pandemic [1,2], current approved vaccines do not generate sufficient immunity to prevent SARS-CoV-2 infection or to reduce the associated symptoms caused by circulating variants [3,4,5,6,7]. Next-generation vaccines that induce sufficient immunity that fully prevent infection are thus needed. Multiple approaches are being pursued: mucosal vaccines that induce immunity at sites of viral entry [8,9,10,11], broader vaccines that generate antibodies capable of neutralizing divergent strains [12,13,14], and more potent vaccines that induce substantially higher titers [15].

Among the approaches to increase breadth and potency, vaccines based on self-assembling nanoparticles appear to be especially promising [16,17,18,19,20,21,22,23,24,25,26,27,28,29,30,31,32,33,34,35,36,37,38,39]. Such nanoparticle vaccines have multiple advantages. Nanoparticles can mimic the size and structure of pathogens, which can lead to a stronger and more specific immune response and result in improved immune memory and longer-lasting protection [40,41]. The nanoparticle format can help protect the vaccine components from degradation, which can increase the shelf life and ease of storage [42]. Nanoparticle vaccines can also display multiple antigens, inducing immunity to different strains of the same pathogen, or even against different pathogens, with a single nanoparticle [43,44].

In this study, we developed nanoparticle vaccines based on encapsulin (En), a self-assembling protein with 60 protomers [45], which has been used in many ways, including drug delivery, vaccine development, cell labeling, and nano-reactions [13,46,47]. We previously created a stabilized version of En by introducing an interprotomer disulfide bond that covalently crosslinked each of the self-assembling protomers of the encapsulin nanoparticle (EnDS) [48]. Additionally, the SpyTag:SpyCatcher conjugation system [15] was used here to create nanoparticle vaccines comprising EnDS displaying multiple RBD proteins (RBD-EnDS-NPs). We improved the stability of the nanoparticles by incorporating nine stabilizing mutations (RBD-Stab) [49], which also improved expression yields. We added these mutations to RBDs from both ancestor (WA1) and BA.5 strains of SARS-CoV-2 and created WA1, BA.5, and mosaic WA1-BA.5 EnDS displaying stabilized RBD proteins (RBD-Stab-EnDS-NPs), which we assessed for stability, antigenicity, and immunogenicity. While all three of these nanoparticles could elicit extraordinarily high autologous neutralization titers (10,000 ID_50_), the BA.5-containing nanoparticles induced much broader neutralization versus the WA1-containing nanoparticle, a finding of potential significance to the development of a next-generation vaccine against SARS-CoV-2.

## 2. Materials and Methods

### 2.1. Animal

BALB/c mice were purchased from Jackson Laboratories (Bar Harbor, ME, USA), and all animals were housed and cared for following American Association for Accreditation of Laboratory Animal Care standards in accredited facilities.

### 2.2. Cell Line

Expi293 cells (Life Technology, Carlsbad, CA, USA) were cultured in Dulbecco’s Modified Eagle Medium (DMEM)-formulated optimal cell growth medium containing 12% inactivated fetal bovine serum (FBS) and 100 U/mL streptomycin–penicillin (ABI Scientific, Sterling, VA, USA) at 37 °C and 5% CO_2_.

### 2.3. Protein Production and Purification

The amino acid sequences of protein constructs are listed in Table S1. The RBD-SpyT proteins were constructed as a fusion protein with an HRV-3C cleavable single-chain Fc purification tag. For protein expression, 3 mL Turbo293 transfection reagent (Speed BioSystems, Gaithersburg, MD, USA) and 50 mL Opti-MEM medium (Life Technology, Carlsbad, CA, USA) were mixed and incubated at room temperature for 5 min. In a separate tube, 1 milligram plasmid DNA was also diluted in 50 mL Opti-MEM, then combined with the Turbo293 mix. The transfection mix was incubated at room temperature for 15 min and then added to 800 mL of Expi293 cells at 2.5 million cells/mL. The culture was placed into an incubation shaker at 37 °C with 9% CO_2_ and 120 rpm shaking. On day 2, 100 mL of Expi293 expression medium was added. Supernatants were harvested on day 5 by centrifugation. Filtered supernatant of RBD-SpyT was passed through a Protein A column. The column was washed and resuspended with PBS (Phosphate Buffer Saline). RBD-SpyT was cleaved off the column by incubating with HRV3C. Final purification was achieved via size exclusion chromatography on a Superpose 6 Increase 10/300 GL column (Cytiva, Marlborough, MA, USA) in PBS buffer.

The gene of Encapsulin-DS with SpyCatcher (EnDS-SpyT) was codon optimized and cloned into a pET11a vector (Novagen, Madison, WI, USA). The plasmid was transformed into One Shot™ BL21(DE3) (Thermo Fisher, Norristown, PA, USA) cells. The cells were grown in LB media containing ampicillin at 37 °C to an OD of 0.6 before induction with 0.5 mM IPTG overnight at 18 °C. The harvested bacterial cells were lysed by sonication, and the supernatant was heated at 56 °C for 15 min. The supernatant was clarified by centrifuge, and saturated (NH_4_)_2_SO_4_ solution was added to the final 20% saturation. The precipitations were harvested and re-suspended in PBS, and endotoxin was removed using TX114 treatment [50]. EnDS nanoparticles were finally purified using a HiPrep™ 26/60 Sephacryl S-500 HR column (Cytiva, Marlborough, MA, USA) in PBS with a flow rate of 1.5 mL/min.

### 2.4. EnDS-SpyC and RBD-SpyT Conjugation

EnDS-SpyC and RBD-SpyT or RBD-Stab-SpyT components were combined at 1:1.5 molar ratio (Encapsulin protomer:RBD) in PBS and incubated at 4 °C overnight. Size exclusion chromatography was carried out to purify the conjugated nanoparticles on a Superose 6 Increase 10/300 GL column (Cytiva, Marlborough, MA, USA) in PBS. Conjugation was confirmed by negative-stain EM and SDS-PAGE under reducing conditions using 4–12% NuPAGE gel and NuPAGE MES SDS Running buffer (Invitrogen, Carlsbad, CA, USA).

### 2.5. Negative-Stain Electron Microscopy (EM)

Negative stain grids were prepared at a sample concentration of 0.02–0.05 mg/mL in buffer containing 10 mM HEPES, pH 7, and 150 mM NaCl. A 4.7 μL drop of the diluted sample was applied to a glow-discharged carbon-coated copper grid for approximately 15 s and then removed using blotting paper. The grid was washed three times with 4.7 μL buffer. Three 4.7 μL drops of 0.75% uranyl formate were applied to the grid-adsorbed proteins and blotted with filter paper. Micrographs were collected using SerialEM [51] on an FEI Tecnai T20 electron microscope (software version Tecnain 4.6.4) (FEI, Hillsboro, OR, USA) operated at 200 kV and equipped with an Eagle CCD camera or using EPU on a ThermoFisher Talos F200C electron microscope (software version Talos 1.15.4) (Thermo Fisher Scientific, Waltham, MA, USA) operated at 200 kV and equipped with a Ceta CCD camera with pixel size 0.44 and 0.25 nm, respectively. Particles were picked automatically using in-house written software version 1.02 (Y.T., unpublished) for reference-free 2D classification using Relion 1.4 and SPIDER 26.06 [52,53].

### 2.6. Preparation of SARS-CoV-2 Antibodies

Codon-optimized antibody heavy-chain and light-chain sequences of CB6 and LY-Cov1404 were synthesized and cloned into a VRC8400-based IgG1 vector and co-expressed by transient transfection to Expi293 cells (Thermo Fisher Scientific, Waltham, MA, USA) according to the manufacturer’s recommendation. Briefly, 50 μg plasmid-encoding heavy-chain genes and 50 μg plasmid-encoding light-chain genes were mixed with 300 μL of Turbo293 transfection reagent (SPEED BioSystems, Gaithersburg, MD, USA) for 15 min, added to 100 mL of cells at a concentration of 2.5 × 10^6^/mL, and incubated in a shaker incubator at 120 rpm and 37 °C under 9% CO_2_. On days 1 and 3 post transfection, an enriched feed medium, AbBooster Antibody Expression Enhancer for suspension cells (ABI Scientific, Sterling, VA, USA), was added into the culture at a 10% culture volume. After 5 days post transfection, cell culture supernatant was harvested and loaded onto a Protein A column (Thermo Fisher Scientific, Waltham, MA, USA). The antibody was eluted using IgG Elution Buffer (Thermo Fisher Scientific, Waltham, MA, USA) and brought to neutral pH (7.0) with 1 M Tris-HCl (pH 8.0). Eluted antibodies were dialyzed against PBS overnight before use.

### 2.7. Bio-Layer Interferometry

Bio-Layer Interferometry (BLI) was carried out with an octet instrument (Sartorius, Goettingen, Germany). Anti-human IgG Fc Capture (AHC) biosensors were used to capture human antibodies. Then the sensors were dipped into a dilution series of immunogens including RBD and RBD-EnDS-NP proteins.

Serum antibody binding with RBD immunogens was also tested with Bio-Layer Interferometry. Briefly, Ni-NTA sensors were used to capture the WA1 or BA.5 RBD proteins, and the sensors were then dipped into 100-fold diluted serum in PBS.

### 2.8. Mouse Immunization

Immunizations were carried out using six-week-old female BALB/cJ mice (Jackson Laboratories, Bar Harbor, ME, USA) by intramuscular inoculation with Sigma Adjuvant System (10 mice in each group) at weeks 0 and 3. Serum was collected from mouse tail 2 weeks post-prime and post-boost for evaluation of antibody responses as detailed hereafter. Mice were kept on study until at least week 8.

### 2.9. Lentivirus-Based Pseudovirus Neutralization Assay

The pseudovirus neutralization assay was performed as described previously [54,55]. SARS-CoV-2 pseudoviruses were obtained by co-transfecting of plasmids encoding codon-optimized CMV/R-SARSCoV-2 spike Wuhan-1 (WA1) or BA.5 strain and luciferase reporter, human transmembrane protease serine 2 (TMPRSS2) [56], and lentivirus backbone into HEK293T/17 cells (ATCC #CRL-11268), as previously described [57]. Serum was heat-inactivated and mixed with the pseudovirus. The mixture was incubated at 37 °C and then added to 293T cells. After 72 h, cells were lysed and luciferase activity was measured. Reading with uninfected cells was set as 100% neutralization, while cells infected with only pseudovirus was set as 0% neutralization. ID_50_/ID_80_ titers of serum were determined using a log (agonist) vs. normalized response (variable slope) nonlinear function in Prism v9 (GraphPad 9.3.1 (471)).

### 2.10. Statistical Analysis

Statistical comparisons among the groups were conducted using the Kruskal–Wallis test. Following a significant Kruskal–Wallis, a post hoc pairwise multiple comparisons Dunn’s test was employed to identify the groups that are different. To account for multiple hypotheses testing, a Bonferroni correction was applied. *p* values less than 0.05 were considered significant (* *p* < 0.05; ** *p* < 0.01; *** *p* < 0.001; **** *p* < 0.0001).

## 3. Results

### 3.1. Design and Characterization of EnDS Nanoparticles Displaying WA1 or BA.5 RBDs

**Figure 1:**
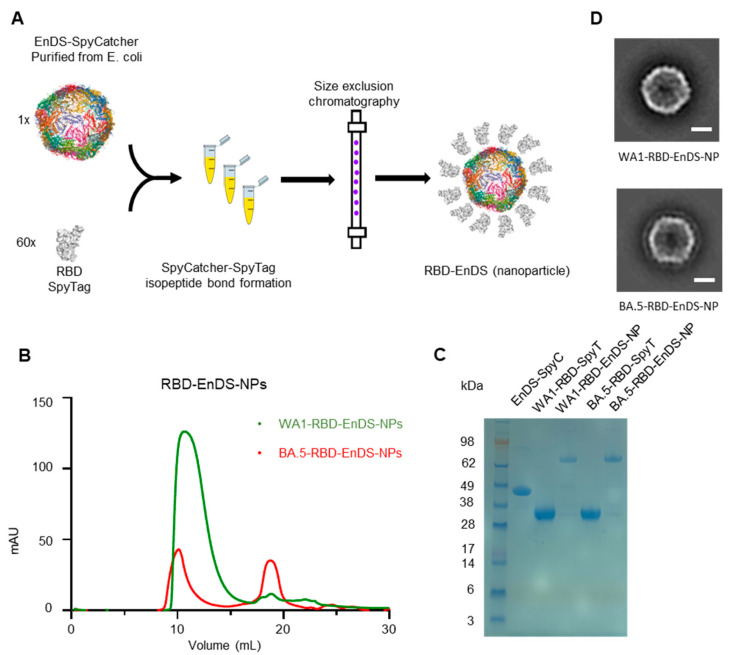
Generation and characterization of Encapsulin-DS (EnDS) nanoparticles displaying RBDs. (**A**) Schematic of the nanoparticle synthesis progress. (**B**) SEC profiles of purified WA1-RBD-EnDS-NPs (green) and BA.5-RBD-EnDS-NP (red). (**C**) SDS-PAGE of EnDS-SpyC, RBD-SpyT and conjugated products. (**D**) Transmission electronic microscopy images of negative-stained nanoparticles: WA1-RBD-EnDS-NP (top) and BA.5-RBD-EnDS-NP (bottom). The scale bars indicate 10 nm.

To produce RBDs, from strains WA1 and BA.5, displayed on the surface of EnDS nanoparticles, we used the SpyTag:SpyCatcher conjugation system [15] (Figure 1A). For conjugation, both RBD proteins were fused with a SpyTag (SpyT) protein on the N terminus, and a SpyCatcher (SpyC) protein was fused to the EnDS nanoparticle. SEC profiles of both RBD proteins can be found in Figure S2. Conjugation occurred at room temperature by mixing the RBD-fused SpyT with the EnDS-fused SpyC—and the resultant mixture was subjected to size exclusion chromatography (Figure 1B). SDS-PAGE analysis (Figure 1C) showed the appearance of a higher molecular weight band (~70 kDa), which corresponded to the combination of EnDS-SpyC (43 kDa) and RBD-SpyT (27 kDa). Transmission electron microscopy (TEM) images of the negative-stained nanoparticles (Figure 1D) revealed only vague small protrusions on the surface of EnDS, as the small size of RBD is close to the resolution limit of TEM.

### 3.2. Antigenic Analysis and Stability Assessment for EnDS Nanoparticles Displaying WA1- and BA.5-RBDs

**Figure 2:**
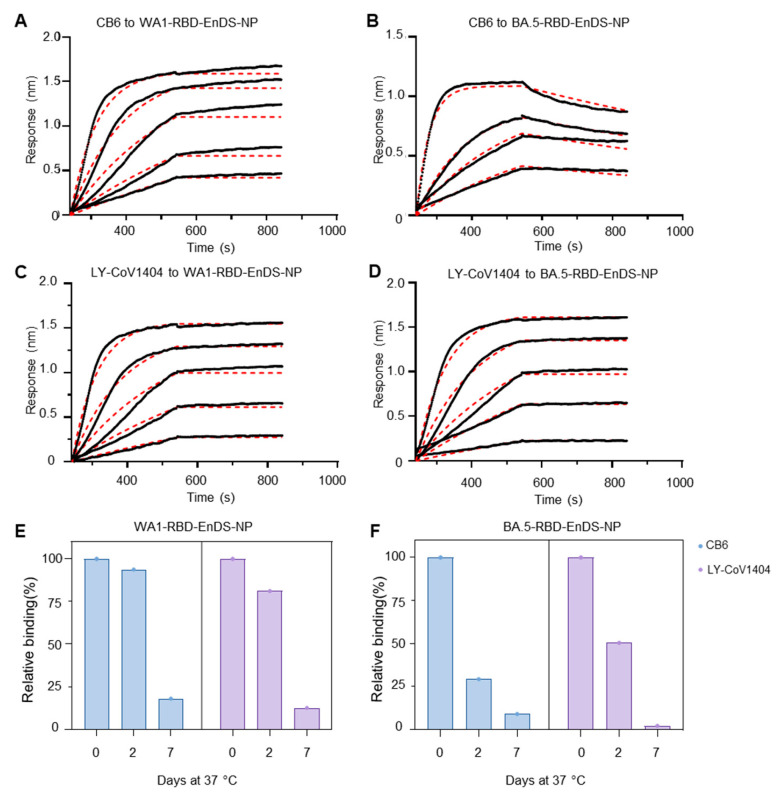
RBD-nanoparticles display good antigenicity but poor stability. (**A**–**D**) BLI sensorgrams are shown in black with fitting provided with dotted red lines. (**A**) BLI analysis of CB6 binding to WA1-RBD-EnDS-NP. (**B**) BLI analysis of CB6 binding to BA.5-RBD-EnDS-NP. (**C**) BLI analysis of LY-Cov1404 binding to WA1-RBD-EnDS-NP. (**D**) BLI analysis of LY-Cov1404 binding to BA.5-RBD-EnDS-NP. (**E**) WA1-RBD-EnDS-NP stability study. The nanoparticle was incubated at 37 °C and assessed for binding to antibodies CB6 and LY-Cov1404 by BLI. (**F**) BA.5-RBD-EnDS-NP stability study. The nanoparticle was incubated at 37 °C and assessed for binding to CB6 and LY-Cov1404 by BLI. (Data shown in panels (**E**,**F**) are from single measurements).

We next evaluated the antigenic characteristics of the two RBD-displaying EnDS nanoparticles with two monoclonal antibodies: CB6 [58], a WA1 RBD -specific antibody that does not bind BA.5 (Figure S3A,B); while another antibody, LY-Cov1404 [59], which binds both WA1 and BA.5 SARS-CoV-2 variants. Octet binding data revealed an increased on-rate for CB6 and LY-Cov1404 antibodies to the WA1-RBD-EnDS-NP (Figure 2A,C and Figure S3A,C). Similarly, CB6 and LY-Cov1404 also showed an increased on-rate to BA.5-RBD-EnDS-NP (Figure 2B,D and Figure S3B,D). We also observed a decreased off-rate to antibody CB6 for the BA.5-RBD-EnDS-NP group (Figure 2B) as CB6 showed no binding to BA.5-RBD alone (Figure S3B), indicative of the successful multimerization of BA.5-RBD on the EnDS nanoparticles.

Having produced both WA1 and BA.5-RBD-EnDS nanoparticles, we next tested the nanoparticle stability at 37 °C. These tests utilized Octet-based measurements of antibody binding after incubation for 2 and 7 days at 37 °C. Notably, we observed substantial lower binding in both nanoparticle groups at day 7, especially for the BA.5-RBD-EnDSEnDS-NP (Figure 2E,F). After 7 days of incubation at 37 °C, BA.5-RBD-EnDS-NP lost ~90% of its antibody binding (Figure 2F).

### 3.3. Development of Stabilized Nanoparticle Immunogens and Characterization

**Figure 3:**
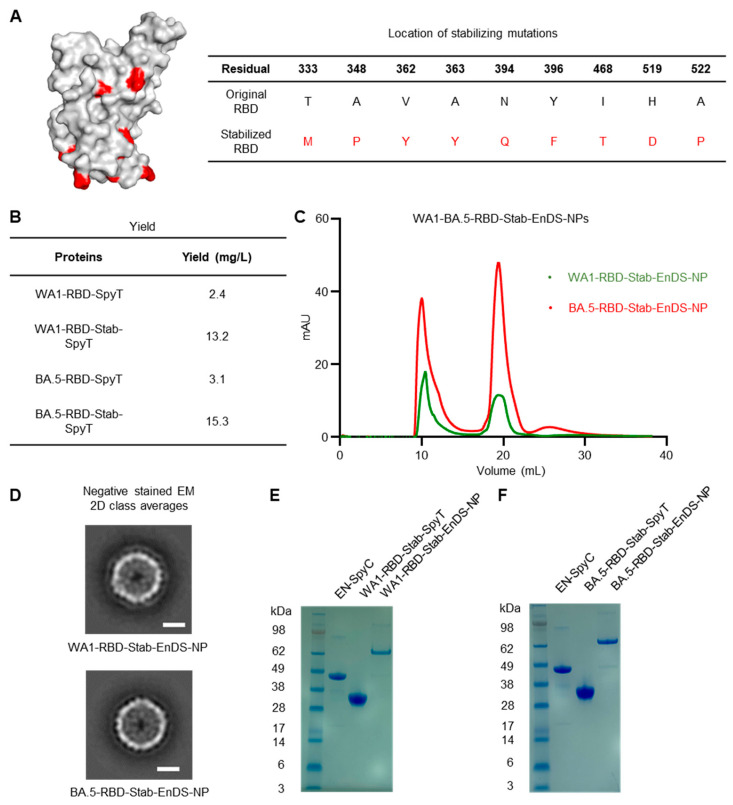
Stabilized RBD-EnDS nanoparticles produced with increased yield. (**A**) Stabilized RBD with amino acid mutations highlighted in red. (**B**) Protein production yield. (**C**) SEC profiles of EnDS nanoparticles display stabilized RBDs: WA1-RBD-Stab-EnDS-NP (green), and BA.5-RBD-Stab-EnDS-NP (red). (**D**) Transmission electronic microscopy images of the negatively stained nanoparticles: WA1-RBD-Stab-EnDS-NP (top) and BA.5-RBD-Stab-EnDS-NP (bottom). The scale bars indicate 10 nm. (**E**) SDS-PAGE of WA1-RBD-Stab-EnDS-NP and its components. (**F**) SDS-PAGE of BA.5-RBD-Stab-EnDS-NP and its components.

To increase the stability of the RBDs, we introduced nine amino acid mutations (Figure 3A and Figure S1), which have been identified using a generalizable computational strategy named SPEEDesign and shown to improve neutralizing antibody titers, production yield, and thermostability of vaccine antigens [16,49,60], and which did not overlap with changes between WA-1 and BA.5. SEC profiles of both stabilized RBD proteins can be found in Figure S2. Notably, we observed ~five-fold higher yield with stabilized version of both WA-1 and BA.5 RBDs (named WA1-RBD-Stab-SpyT and BA.5-RBD-Stab-SpyT, respectively) (Figure 3B). We used SpyTag:SpyCatcher conjugation to generate EnDS nanoparticles displaying stabilized RBDs (named WA1-RBD-Stab-EnDS-NP and BA.5-RBD-Stab-EnDS-NP). From the SEC profile (Figure 3C), we observed a sharper nanoparticle peak of around 10 mL compared with the non-stabilized version. Negative-stain TEM images (Figure 3D) confirmed the integrity of stabilized RBD-EnDS nanoparticles, and SDS-PAGE analysis revealed the conjugated product to have the expected molecular weight (Figure 3E,F).

### 3.4. Stabilized WA1 and BA.5 Nanoparticles Show Superior Antigenicity and Improved Stability at 37 °C

**Figure 4:**
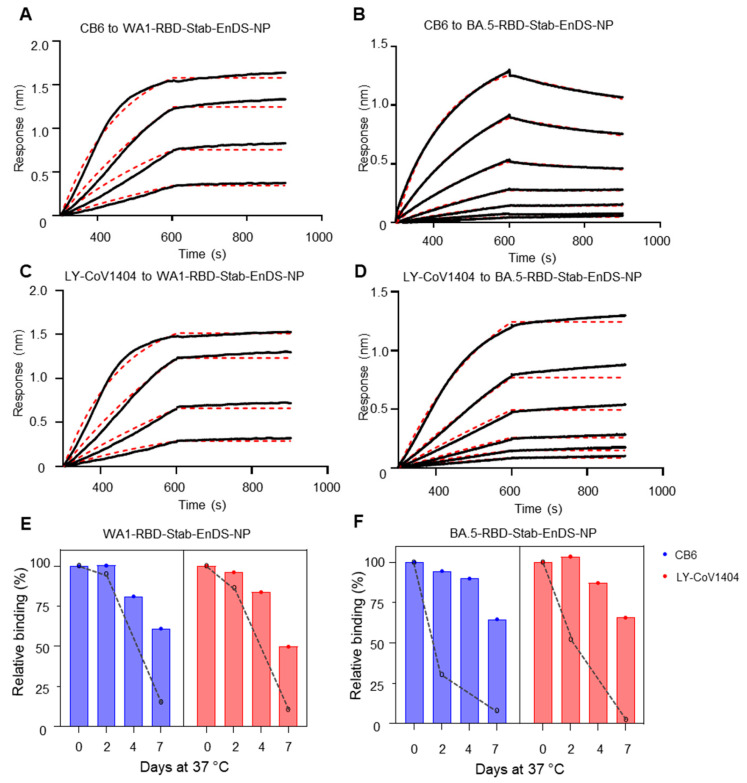
Stabilized WA1 and BA.5 nanoparticles show superior antigenicity and improved stability at 37 °C. (**A**–**D**) BLI sensorgrams are shown in black with fitting provided with dotted red lines. (**A**) BLI binding analysis of antibody CB6 with WA1-RBD-Stab-EnDS-NP. (**B**) BLI binding analysis of antibody CB6 with BA.5-RBD-Stab-EnDS-NP. (**C**) BLI binding analysis of antibody LY-CoV1404 with WA1-RBD-Stab-EnDS-NP. (**D**) BLI binding analysis of antibody LY-CoV1404 with BA.5-RBD-Stab-EnDS-NP. (**E**) WA1-RBD-Stab-EnDS-NP thermo-stability study. The nanoparticle was incubated at 37 °C for the indicated time and analyzed for the remaining binding capacity with antibodies CB6 and LY-CoV1404 by BLI. Black dashed lines show the relative binding signals of the non-stabilized RBD nanoparticle. (**F**) BA.5-RBD-Stab-EnDS-NP thermo-stability study. The nanoparticle was assessed as described in (**E**). Black dashed lines show the relative binding signals of the non-stabilized RBD nanoparticle. (Data shown in panels (**E**,**F**) are from single measurements).

By using the same method described above for WA1-RBD-EnDS-NP and BA.5-RBD-EnDS-NP, we assessed the antigenicity of nanoparticles displaying stabilized RBDs, WA1-RBD-Stab-EnDS-NP and BA.5-RBD-Stab-EnDS-NP, by dipping antibody-coupled sensors into various concentrations of nanoparticle solutions. Similar to the observations above, we observed an increased on-rate for the WA1-RBD-Stab-EnDS-NP binding to CB6 or LY-Cov1404 antibodies (Figure 4A,C). While there was no binding between CB6 and BA.5-RBD-Stab (Figure S4B), there was substantial CB6 binding to BA.5-RBD-Stab-EnDS-NP with an on-rate and off-rate comparable with its binding to BA.5-RBD-EnDS-NP (Figure 4B). Additionally, BA.5-RBD-Stab-EnDS-NP also showed an increased on-rate and decreased off-rate to LY-Cov1404 compared with binding to monomeric RBD (Figure 4D and Figure S4, and Table S2).

Most importantly, the incorporation of stabilized RBD proteins onto nanoparticles showed substantially enhanced stability compared with the nanoparticles displaying native RBD proteins. Both WA1- and BA.5-RBD-Stab-EnDS-NPs showed more than 60% binding reactivity to be retained after 7 days of incubation at 37 °C (Figure 4E,F). Relative to the non-stabilized version nanoparticles, the BA.5-RBD-Stab-EnDS-NP had more improvement in stability than WA1-RBD-Stab-EnDS-NP (Figure 4E,F, comparing bars with dashed lines).

### 3.5. Immunization of Stabilized RBD-EnDS Nanoparticles Elicited Strong Anti-SARS-CoV-2 Pseudovirus Neutralizing Responses in Mice

**Figure 5:**
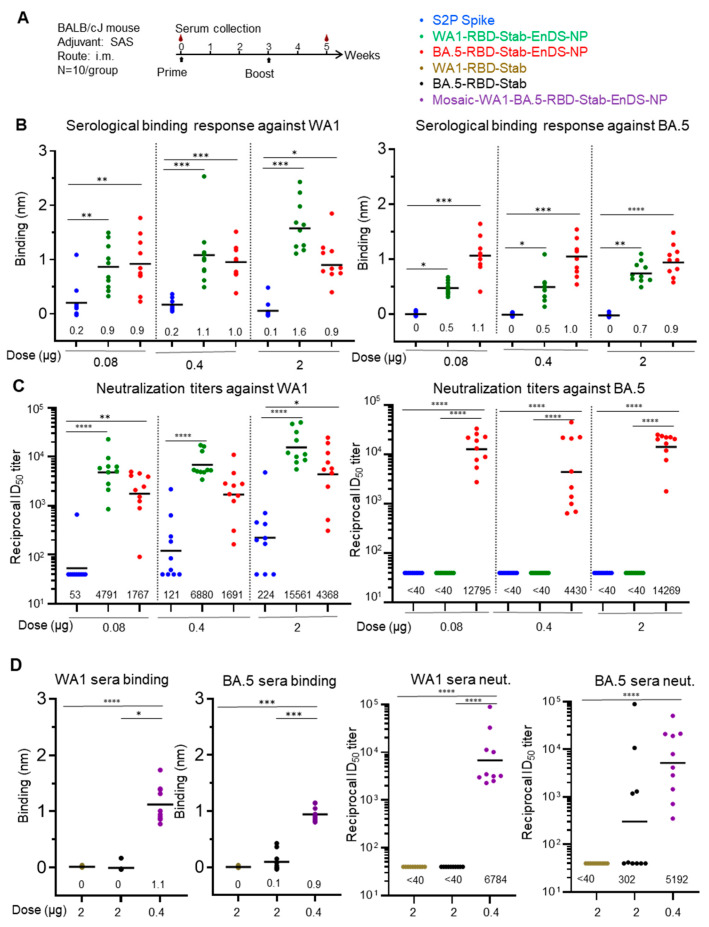
Immunization of stabilized RBD-EnDS nanoparticles elicited strong anti-SARS-CoV-2 pseudovirus neutralizing responses in mice. (**A**) Scheme of the protein immunization regimen. The immunization groups are color-coded based on the immunogens used, as shown on the right side of the panel; this color coding is maintained throughout the entire figure. (**B**) Serological binding response against WA1 and BA.5 RBD from the immunized mouse sera (week 5). (**C**) Anti-SARS-CoV-2 WA1 and BA.5 pseudovirus neutralization ID_50_ titers from EnDS NPs and S2P-Spike immunizations (week 5). (**D**) Anti WA1/BA.5 RBD serum binding and anti-SARS-CoV-2 WA1 and BA.5 pseudovirus neutralization ID_50_ titers from RBD-Stab proteins and mosaic-WA1-BA.5-RBD-Stab-EnDS-NP (week 5). (*: *p*-value less than 0.05; **: *p*-value less than 0.01; ***: *p*-value less than 0.001; ****: *p*-value less than 0.0001). In panels (**B**–**D**), the numbers above the horizontal axes are geometric mean values of each group.

To assess immunogenicity, we injected mice with two shots of proteins in week 0 and week 3. For groups with two of the nanoparticles, WA1-RBD-Stab-EnDS-NP and BA.5-RBD-Stab-EnDS-NP, and groups with 2-proline stabilized Spike protein from the WA1 strain (S2P Spike), three doses of proteins were included in the immunization (0.08, 0.4, or 2 μg/mouse) (Figure 5A). We also included three additional groups, the WA1-RBD-Stab group (2 μg/mouse), the BA.5-RBD-Stab group (2 μg/mouse), and the mosaic-WA1-BA.5-RBD-Stab-EnDS-NP group (0.4 μg/mouse). The mosaic-WA1/BA.5-RBD-Stab-EnDS-NP was prepared by mixing the same amount of WA1- or BA.5-RBD-Stab-SpyT and incubated with EnDS-SpyC. After incubation, the protein mixture was loaded onto the SEC column to purify the conjugated nanoparticle product from unconjugated nanoparticles and RBD proteins.

We analyzed serum antibody response from mouse sera two weeks after the second immunizations with Octet. The WA1- or BA.5-RBD-Stab protein was loaded onto sensors and dipped into 1:100 diluted sera. All groups with the three doses of S2P showed relatively weak sera binding to WA1 but failed to show any binding to BA.5 RBD (Figure 5B). The WA1-RBD-Stab-EnDS immunized groups showed significantly higher binding to both WA1 and BA.5 RBDs than the S2P groups. Similarly, the BA.5-RBD-Stab-EnDS immunized groups showed significantly higher binding to both WA1 and BA.5 RBDs than the S2P groups. The BA.5-RBD-Stab-EnDS groups exhibited higher binding titers to BA.5 RBD protein than to WA1 RBD, and vice versa, but not statistically significant. The stabilized RBD proteins, WA1-RBD-Stab and BA.5-RBD-Stab, induced very low antibody binding titers, whereas the mosaic-WA1/BA.5-RBD-Stab-EnDS-NP induced significantly higher antibody titers against both WA1 and BA.5 RBD proteins, even with a five-fold lower dose (Figure 5D, left two panels).

Importantly, all nanoparticles elicited extraordinarily high homologous neutralization titers, with the highest exceeding geometric mean titer of 15,000 ID_50_. The S2P spike group showed low neutralization titer even in the highest dose group (2 μg), and in the lowest dose group, only one out of the ten mice showed weak neutralizing response against WA1 strain (Figure 5C blue dots). In the WA1-RBD-Stab-EnDS-NP groups, all three doses elicited high neutralization titer against the WA1 strain; even for the 0.08 μg group, the ID_50_ titer was ~4800. BA.5-RBD-Stab-EnDS-NP in all three doses elicited cross neutralization against WA1 strain with high ID_50_ titers of 1767, 1691, and 4368 for the 0.08, 0.4, and 2 μg groups, respectively (Figure 5C, red dots). The S2P Spike protein (WA1 strain) and the WA1-RBD-Stab-EnDS-NP did not elicit detectable neutralization against the BA.5 strain (Figure 5C green dots). Detectable neutralization was also not observed with the monomeric WA1-RBD-Stab protein, and only 4 out of 10 mice immunized with BA.5-RBD-Stab showed detectable neutralization responses at a 2 μg dose. In contrast, we observed high neutralization titers in all the mice immunized with 0.4 μg mosaic-WA1-BA.5-RBD-Stab-EnDS-NPs, with geometric mean ID_50_ of 6784 against the WA1 strain and 5192 against the BA.5 strain (Figure 5D).

**Figure 6:**
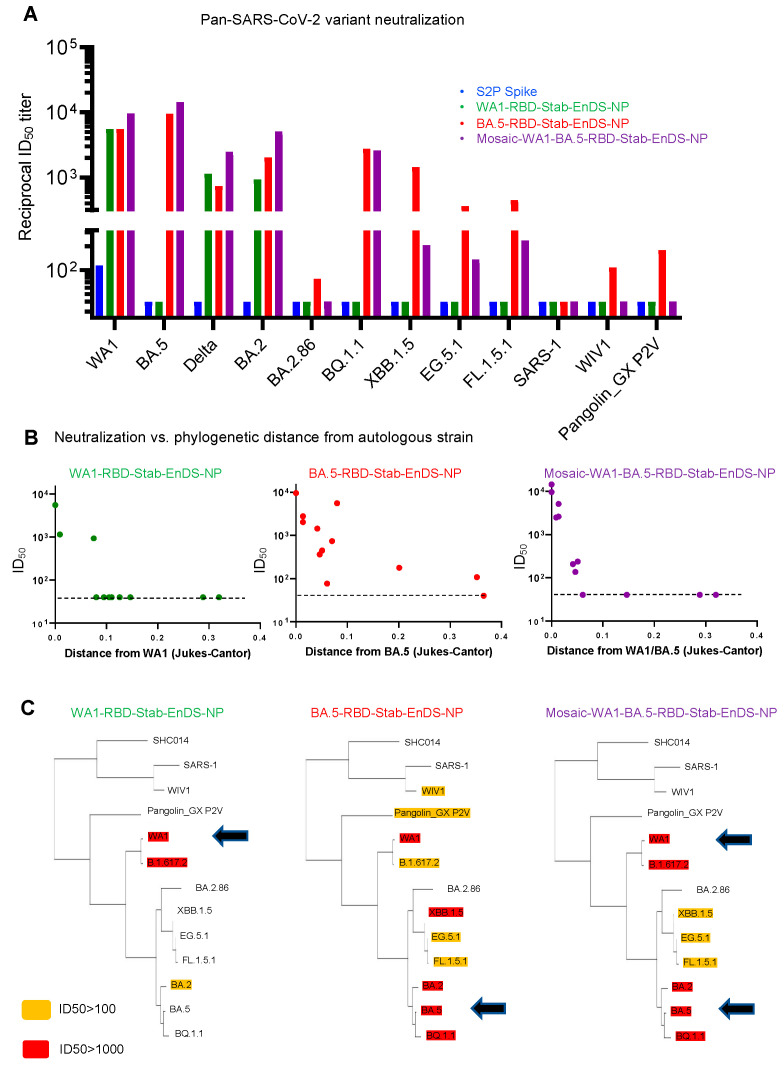
BA.5-RBD-Stab-EnDS-NP elicits broadly neutralizing responses across SARS-CoV-2 variants and diverse beta-coronavirus strains. (**A**) Neutralization assessments of pooled sera from immunized mice at week 5 against a panel of SARS-CoV-2 variants and related beta-coronavirus strains. (**B**) Neutralization titers versus phylogenetic distances of the tested variants/strains from the immunized variant. The dotted line indicates threshold of neutralization detection limit. (**C**) Phylogenetic tree of SARS-CoV-2 variants and related beta-coronavirus strains with neutralization titers indicated by highlight colors. Black arrows mark the autologous variant (or variants).

To provide insight into the elicited neutralization breadth, we assessed the group-pooled sera from the 0.4 μg dose groups immunized with the nanoparticles and S2P spike on a panel of diverse beta-coronaviruses (Figure 6). We chose the 0.4 ug dose group because we had mosaic nanoparticle measurements at this dose. The pooled sera of the S2P spike group only showed weak neutralization against the WA1 strain (Figure 6A). The WA1-RBD-Stab-EnDS-NP group showed high immunization titers against WA1, B.1.617.2 (Delta), and BA.2, but neutralization against other strains was below the limit of detection (<40 ID_50_). The BA.5-RBD-Stab-EnDS-NP and mosaic-WA1-BA.5-RBD-Stab-EnDS-NP groups neutralized substantially more strains, with the BA.5-RBD-Stab-EnDS-NP group being the broadest, with neutralization from only a single strain being below the limit of detection, versus 10 and 4 strains being below the limit of detection for WA-1 and mosaic-WA1-BA.5 nanoparticles, respectively.

We plotted neutralization titers versus phylogenetic distances between the strains/variants and found that neutralization titers dropped quickly as the phylogenetic distance increased (Figure 6B). The BA.5-RBD-Stab group showed much higher retention of neutralization titers across a wide range of variants, including XBB.1.5 and the current spreading variants including EG.5.1 and BQ.1.1. Broad neutralization was also observed in the mosaic nanoparticle group, though the drop-off with respect to phylogenetic distance was more pronounced than with the BA.5 nanoparticle (Figure 6B,C).

## 4. Discussion

The nanoparticle vaccine, SKYCovione, utilizing WA1 RBDs displayed on a self-assembling two-component nanoparticle [14] was approved last year by the Korean Ministry of Food and Drug Safety for use in individuals 18 years of age and older [61,62]. While this vaccine generates three-fold higher titers in humans than the Oxford–AstraZeneca COVID-19 vaccine [63], our results with WA1 RBD suggest that it may not generate high titer neutralization against current circulating strains. Similar results may hold true for other approved RBD vaccines, such as Corbevax, Abdala, and ZF2001, as well as those in clinical trials, such as mRNA-1283 [64,65,66,67]. Instead, our results indicate that nanoparticles displaying stabilized RBDs from the BA.5 strain should be used, as these RBDs induce substantially higher neutralization titer against current circulating strains.

The neutralization elicited by the BA.5-RBD-Stab-EnDS-NP against current—and divergent—strains was quite remarkable, with observable neutralization against 12 of 13 tested strains, all except SARS-1. The observed elicited breadth suggests the BA.5-RBD-Stab-EnDS-NPs should be able to elicit neutralization titers against evolving strains for the next few years. Whether the murine titers and breadth that we observed from the BA.5-RBD-Stab-EnDS-NP described here will be also observed in other test species and in humans remains to be determined. Nonetheless, our results indicate specific strains of SARS-CoV-2 may be able to generate much greater neutralization breadth than others, highlighting the importance of choosing the appropriate RBD sequence for nanoparticle display.

The nine amino acid mutations identified using SPEEDesign have now improved WA-1, Beta, and BA.5 RBD vaccines in both monomeric and nanoparticle formats, suggesting that these mutations are broadly applicable [49]. This is consistent with the fact that these stabilizing mutations do not overlap with known variant changes. SPEEDesign fixes known neutralizing epitopes in antigens and classifies the remaining amino acids into distinct categories that are allowed to vary to different extents, resulting in enhanced immunogens [16,49,60]. It is not entirely clear what degraded the non-stabilized RBD, but we observed no proteolytic digestion, so degradation was likely related to unfolding, with the mutations in RBD improving production yields and thermostability, and likely contributing to the potent and broad response elicited by the BA.5-RBD-Stab-EnDS-NP.

By using the SpyTag:SpyCatcher conjugation system, we were able to design and engineer nanoparticles to display specific antigens or combinations of antigens. It will be of interest to test specifically the BA.5-RBD-Stab-EnDS-NP vaccine for its ability to boost mucosal responses. Relevant to this, the SpyTag:SpyCatcher conjugation system could be used to add albumin, as nanoparticles incorporating albumin may have enhanced translocation abilities, leading to enhanced immune responses when delivered nasally, as mucosal vaccination is likely critical to enable immune responses that prevent infection. While the plug-and-display [15] nature of this system lends to its versatility, it remains to be seen if this system can be used clinically to provide vaccines utilizing RBDs that elicit high neutralization breadth and potency against circulating strains.

## 5. Conclusions

Our study demonstrates nanoparticles displaying native SARS-CoV-2 RBDs to have stability issues. We further demonstrate that introducing nine site-specific RBD mutations increased both nanoparticle production yield and nanoparticle stability at 37 °C, and—upon immunization—these nanoparticles could induce high titer autologous neutralization responses in mice. While the WA1-displaying nanoparticles induced neutralization breadth that extended only to phylogenetically similar strains (e.g., Delta), the BA.5-containing nanoparticles elicited neutralization that extended much broader, from the SARS-CoV-2 ancestor strain to most variants of concerns (VOC) and even to more divergent sarbecoviruses (e.g., WIV1 and Panngolin_GX). The unexpected breadth of BA.5 RBD-Stab-displaying nanoparticles suggests they may be strong next-generation SARS-CoV-2 candidate vaccines.

## Data Availability

The resources and materials used in the manuscript are available from the corresponding author upon request. Any such requests should be directed to and will be fulfilled by Tongqing Zhou (tzhou@nih.gov) or Peter D. Kwong (pdkwong@nih.gov).

## Supplementary Materials

The following supporting information can be downloaded at: https://www.mdpi.com/article/10.3390/vaccines12010037/s1, Table S1. Amino acid sequences of constructs for protein expression. Table S2. Octet binding data of different immunogens with CB6 or LY-Cov1404 antibodies. Table S3. Phylogenetic distance between RBDs. Figure S1. Amino acid sequence alignment of different RBD constructs. Figure S2. High-purity RBD-SpyT proteins produced for nanoparticle preparation. Figure S3. Antigenic analysis of different RBD proteins. Figure S4. Antigenic analysis of different RBD proteins and Mosaic-WA1-BA.5-Stab-EnDS-NP. Figure S5. Immunization of stabilized RBD-EnDS nanoparticles elicited strong anti-SARS-CoV-2 pseudovirus neutralizing responses in mice. Figure S6. Uncropped SDS-PAGE images and densitometry readings of each lane.
