# Supplementary material for: Extraordinary Titer and Broad Anti-SARS-CoV-2 Neutralization Induced by Stabilized RBD Nanoparticles from Strain BA.5
Source: Vaccines (Basel). 2023 Dec 28;12(1):37. doi: 10.3390/vaccines12010037 (PMC10821209; doi:10.3390/vaccines12010037)
Supplement: Supplementary file 1 [file vaccines-12-00037-s001.zip › vaccines-2767962-supplementary.pdf]

Table S1. Amino acid sequences of constructs for protein expression. scFc sequences highlighted in blue, and RBD/RBD-Stab-SpyT sequences highlighted in purple.

Construct name	Amino acid sequence
Encapsulin-DS-SpyC	MEFLKRSFAPLTEKQWQEIDNRAREIFKTQLYGRKFVDVEGGGGGHHHHHHGGGGGPYGWEYAAHP<br>LCEVEVLSDENEVVKWGLRKSLPLIELRATFTLLWELDNLECGKPNVDLSSLEETVRKVAEFEDEVIFRG<br>CEKSGVKGLLSFEERKIECGSTPKDLLEAIVRALSIFSKDGIEGPYTLVINTDRWINFLKEEAGHYPLEKR<br>VEECLRGGKIITTPRIEDALVVSERGGDFKLILGQDLSIGYEDREKDAVRLFITETFTMLLKFGSGSGSVT<br>TLSGLSGEQGPSGDMTTEEDSATHIKFSKRDEDGRELAGATMELRDSSGKTISTWISDGHVKDFYLYP<br>GKYTFVETAAPDGYEVATAITFTVNEQGQVTVNGEATKGDAHTGSSGS
WA1-RBD-SpyT	MGWSCIILFLVATATGVHSAPELLGGPSVFLFPPKPKDTLMISRTPEVTCVVVDVSHEDPEVKFNWYVD<br>GVEVHNAKTKPREEQYNSTYRVVSVLTVLHQDWLNGKEYKCKVSNKALPAPIEKTISKAKGQPREPQV<br>YTLPPSRDELTKNQVSLYCLVKGFYPSDIAVEWESNGQPENNYKTTPPVLDSDGSFFLYSKLTVDKSR<br>WQQGNVFSCSVMHEALHNHYTQKSLSLSPGKGGGGSGGGGSGGGGSGGGGSAPELLGGPSVFLFP<br>PKPKDTLMISRTPEVTCVVVDVSHEDPEVKFNWYVDGVEVHNAKTKPREEQYNSTYRVVSVLTVLHQD<br>WLNGKEYKCKVSNKALPAPIEKTISKAKGQPREPQVYTLPPSRDELTKNQVSLTCLVKGFYPSDIAVEW<br>ESNGQPENNYKTTPPVLDSDGSFFLTSKLTVDKSRWQQGNVFSCSVMHEALHNHYTQKSLSLSPGKG<br>GSGGGGSGGLEVLFQGPAHIVMVDAYKPTKGGSGGSGGRVQPTESIVRFPNITNLCPFGEVFNATRFA<br>SVYAWNRKRISNCVADYSVLYNSASFSTFKCYGVSPTKLNDLCFTNVYADSFVIRGDEVRQIAPGQTGK<br>IADYNYKLPDDFTGCVIAWNSNNLDSKVGGNYNYLYRLFRKSNLKPFERDISTEIYQAGSTPCNGVEGF<br>NCYFPLQSYGFQPTNGVGYQPYRVVVLSFELLHAPATVCGPKKSTNLVKNK
BA.5-RBD-SpyT	MGWSCIILFLVATATGVHSAPELLGGPSVFLFPPKPKDTLMISRTPEVTCVVVDVSHEDPEVKFNWYVD<br>GVEVHNAKTKPREEQYNSTYRVVSVLTVLHQDWLNGKEYKCKVSNKALPAPIEKTISKAKGQPREPQV<br>YTLPPSRDELTKNQVSLYCLVKGFYPSDIAVEWESNGQPENNYKTTPPVLDSDGSFFLYSKLTVDKSR<br>WQQGNVFSCSVMHEALHNHYTQKSLSLSPGKGGGGSGGGGSGGGGSGGGGSAPELLGGPSVFLFP<br>PKPKDTLMISRTPEVTCVVVDVSHEDPEVKFNWYVDGVEVHNAKTKPREEQYNSTYRVVSVLTVLHQD<br>WLNGKEYKCKVSNKALPAPIEKTISKAKGQPREPQVYTLPPSRDELTKNQVSLTCLVKGFYPSDIAVEW<br>ESNGQPENNYKTTPPVLDSDGSFFLTSKLTVDKSRWQQGNVFSCSVMHEALHNHYTQKSLSLSPGKG<br>GSGGGGSGGLEVLFQGPAHIVMVDAYKPTKGGSGGSGGRVQPTESIVRFPNITNLCPFDEVFNATRFA<br>SVYAWNRKRISNCVADYSVLYNFAPFFAFKCYGVSPTKLNDLCFTNVYADSFVIRGNEVSQIAPGQTGN<br>IADYNYKLPDDFTGCVIAWNSNKLDSKVGGNYNYRYRLFRKSNLKPFERDISTEIYQAGNKPCNGVAGV<br>NCYFPLQSYGFRPTYGVGHQPYRVVVLSFELLHAPATVCGPKKSTNLVKNK
WA1-RBD-Stab-SpyT	MGWSCIILFLVATATGVHSAPELLGGPSVFLFPPKPKDTLMISRTPEVTCVVVDVSHEDPEVKFNWYVD<br>GVEVHNAKTKPREEQYNSTYRVVSVLTVLHQDWLNGKEYKCKVSNKALPAPIEKTISKAKGQPREPQV<br>YTLPPSRDELTKNQVSLYCLVKGFYPSDIAVEWESNGQPENNYKTTPPVLDSDGSFFLYSKLTVDKSR<br>WQQGNVFSCSVMHEALHNHYTQKSLSLSPGKGGGGSGGGGSGGGGSGGGGSAPELLGGPSVFLFP<br>PKPKDTLMISRTPEVTCVVVDVSHEDPEVKFNWYVDGVEVHNAKTKPREEQYNSTYRVVSVLTVLHQD<br>WLNGKEYKCKVSNKALPAPIEKTISKAKGQPREPQVYTLPPSRDELTKNQVSLTCLVKGFYPSDIAVEW<br>ESNGQPENNYKTTPPVLDSDGSFFLTSKLTVDKSRWQQGNVFSCSVMHEALHNHYTQKSLSLSPGKG<br>GSGGGGSGGLEVLFQGPAHIVMVDAYKPTKGGSGGSGGRVQPTESIVRFPNIMNLCPFGEVFNATRF<br>PSVYAWNRKRISNCYYDYSVLYNSASFSTFKCYGVSPTKLNDLCFTQVFADSFVIRGDEVRQIAPGQTG<br>KIADYNYKLPDDFTGCVIAWNSNNLDSKVGGNYNYLYRLFRKSNLKPFERDTSTEIYQAGSTPCNGVEG<br>FNCYFPLQSYGFQPTNGVGYQPYRVVVLSFELLDAPPTVCGPKKSTNLVKNK
BA.5-RBD-Stab-SpyT	MGWSCIILFLVATATGVHSAPELLGGPSVFLFPPKPKDTLMISRTPEVTCVVVDVSHEDPEVKFNWYVD<br>GVEVHNAKTKPREEQYNSTYRVVSVLTVLHQDWLNGKEYKCKVSNKALPAPIEKTISKAKGQPREPQV<br>YTLPPSRDELTKNQVSLYCLVKGFYPSDIAVEWESNGQPENNYKTTPPVLDSDGSFFLYSKLTVDKSR<br>WQQGNVFSCSVMHEALHNHYTQKSLSLSPGKGGGGSGGGGSGGGGSGGGGSAPELLGGPSVFLFP<br>PKPKDTLMISRTPEVTCVVVDVSHEDPEVKFNWYVDGVEVHNAKTKPREEQYNSTYRVVSVLTVLHQD<br>WLNGKEYKCKVSNKALPAPIEKTISKAKGQPREPQVYTLPPSRDELTKNQVSLTCLVKGFYPSDIAVEW<br>ESNGQPENNYKTTPPVLDSDGSFFLTSKLTVDKSRWQQGNVFSCSVMHEALHNHYTQKSLSLSPGKG<br>GSGGGGSGGLEVLFQGPAHIVMVDAYKPTKGGSGGSGGRVQPTESIVRFPNIMNLCPFDEVFNATRF<br>PSVYAWNRKRISNCYYDYSVLYNFAPFFAFKCYGVSPTKLNDLCFTQVFADSFVIRGNEVSQIAPGQTG<br>NIADYNYKLPDDFTGCVIAWNSNKLDSKVGGNYNYRYRLFRKSNLKPFERDTSTEIYQAGNKPCNGVA<br>GVNCYFPLQSYGFRPTYGVGHQPYRVVVLSFELLDAPPTVCGPKKSTNLVKNK

Table S2. Octet binding data of different immunogens with CB6 or LY-CoV1404 antibodies.

IgG	Immunogen	KD (M)	KD error	Kon (1/Ms)	Kon error	Koff (1/s)	Koff error
CB6	WA1-RBD-Stab	4.03×10^{-8}	1.35×10^{-9}	1.31×10^{5}	4.15×10^{3}	5.27×10^{-3}	5.58×10^{-5}
CB6	BA5-RBD-Stab	No binding					
CB6	WA1-RBD-Stab-EnDS-NP	$<1.0 \times 10^{-12}$	1.43×10^{-11}	2.72×10^{6}	7.70×10^{4}	$<1.0 \times 10^{-7}$	
CB6	BA.5-RBD-Stab-EnDS-NP	7.84×10^{-10}	1.19×10^{-11}	7.52×10^{5}	5.23×10^{3}	5.89×10^{-4}	7.92×10^{-6}
CB6	Mosaic-WA1-BA.5-Stab-EnDS-NP	$<1.0 \times 10^{-12}$	4.01×10^{-12}	1.99×10^{6}	1.26×10^{4}	$<1.0 \times 10^{-7}$	
LY-CoV1404	WA1-RBD-Stab	$<1.0 \times 10^{-12}$	1.63×10^{-10}	1.93×10^{5}	6.35×10^{3}	$<1.0 \times 10^{-7}$	
LY-CoV1404	BA5-RBD-Stab	$<1.0 \times 10^{-12}$	1.77×10^{-10}	2.13×10^{5}	8.90×10^{3}	$<1.0 \times 10^{-7}$	
LY-CoV1404	WA1-RBD-Stab-EnDS-NP	$<1.0 \times 10^{-12}$	1.27×10^{-11}	3.04×10^{6}	8.48×10^{4}	$<1.0 \times 10^{-7}$	
LY-CoV1404	BA.5-RBD-Stab-EnDS-NP	$<1.0 \times 10^{-12}$	$9.73 \text{ X } 10^{-12}$	1.05×10^{6}	8.32×10^{3}	$<1.0 \times 10^{-7}$	
LY-CoV1404	Mosaic-WA1-BA.5-Stab-EnDS-NP	$<1.0 \times 10^{-12}$	1.43×10^{-12}	3.30×10^{6}	1.14×10^{4}	$<1.0 \times 10^{-7}$	

Table S3. Phylogenetic distance between RBDs.

Strain	WA1	BA.5	WA1/BA.5
WA1	0	0.0802	0
BA.5	0.0802	0	0
Delta	0.0091	0.0704	0.0091
BA.2	0.0753	0.0137	0.0137
BA.2.86	0.1256	0.0607	0.0607
BQ.1.1	0.0951	0.0137	0.0137
XBB.1.5	0.1052	0.0416	0.0416
EG.5.1	0.1102	0.0464	0.0464
FL.1.5.1	0.1102	0.0511	0.0511
SARS-1	0.3197	0.3652	0.3197
WIV1	0.2886	0.352	0.2886
Pangolin_GX P2V	0.1465	0.2008	0.1465

A

```
Consensus           GPAHIVMVDAYKPTKGGSGGSGGRVQPTESIVRFPNIXNLCPFGEVFNATRFXSVYAWNR   60
WA1-RBD-SpyT        .....................................T..............A.......   60
WA1-RBD-Stab-SpyT   .....................................M..............P.......   60

Consensus           KRISNCXXDYSVLYNSASFSTFKCYGVSPTKLNDLCFTXVXADSFVIRGDEVRQIAPGQT  120
WA1-RBD-SpyT        ......VA..............................N.Y...................  120
WA1-RBD-Stab-SpyT   ......YY..............................Q.F...................  120

Consensus           GKIADYNYKLPDDFTGCVIAWNSNNLDSKVGGNYNYLYRLFRKSNLKPFERDXSTEIYQA  180
WA1-RBD-SpyT        ....................................................I.......  180
WA1-RBD-Stab-SpyT   ....................................................T.......  180

Consensus           GSTPCNGVEGFNCYFPLQSYGFQPTNGVGYQPYRVVVLSFELLXAPXTVCGPKKSTNLVK  240
WA1-RBD-SpyT        ...........................................H..A.............  240
WA1-RBD-Stab-SpyT   ...........................................D..P.............  240

Consensus           NK                                                            242
WA1-RBD-SpyT        ..                                                            242
WA1-RBD-Stab-SpyT   ..                                                            242
```

B

```
Consensus           GPAHIVMVDAYKPTKGGSGGSGGRVQPTESIVRFPNIXNLCPFDEVFNATRFXSVYAWNR   60
BA.5-RBD-SpyT       .....................................T..............A.......   60
BA.5-RBD-Stab-SpyT  .....................................M..............P.......   60

Consensus           KRISNCXXDYSVLYNFAPFFAFKCYGVSPTKLNDLCFTXVXADSFVIRGNEVSQIAPGQT  120
WA1-RBD-SpyT        ......VA..............................N.Y...................  120
WA1-RBD-Stab-SpyT   ......YY..............................Q.F...................  120

Consensus           GNIADYNYKLPDDFTGCVIAWNSNKLDSKVGGNYNYRYRLFRKSNLKPFERDXSTEIYQA  180
WA1-RBD-SpyT        ....................................................I.......  180
WA1-RBD-Stab-SpyT   ....................................................T.......  180

Consensus           GNKPCNGVAGVNCYFPLQSYGFRPTYGVGHQPYRVVVLSFELLXAPXTVCGPKKSTNLVK  240
WA1-RBD-SpyT        ...........................................H..A.............  240
WA1-RBD-Stab-SpyT   ...........................................D..P.............  240

Consensus           NK                                                            242
WA1-RBD-SpyT        ..                                                            242
WA1-RBD-Stab-SpyT   ..                                                            242
```

Figure S1. Amino acid sequence alignment of different RBD constructs. (A) WA1-RBD-SpyT and WA1-Stab-RBD-SpyT protein alignment. (B) BA.5-RBD-SpyT and BA.5-Stab-RBD-SpyT protein alignment.

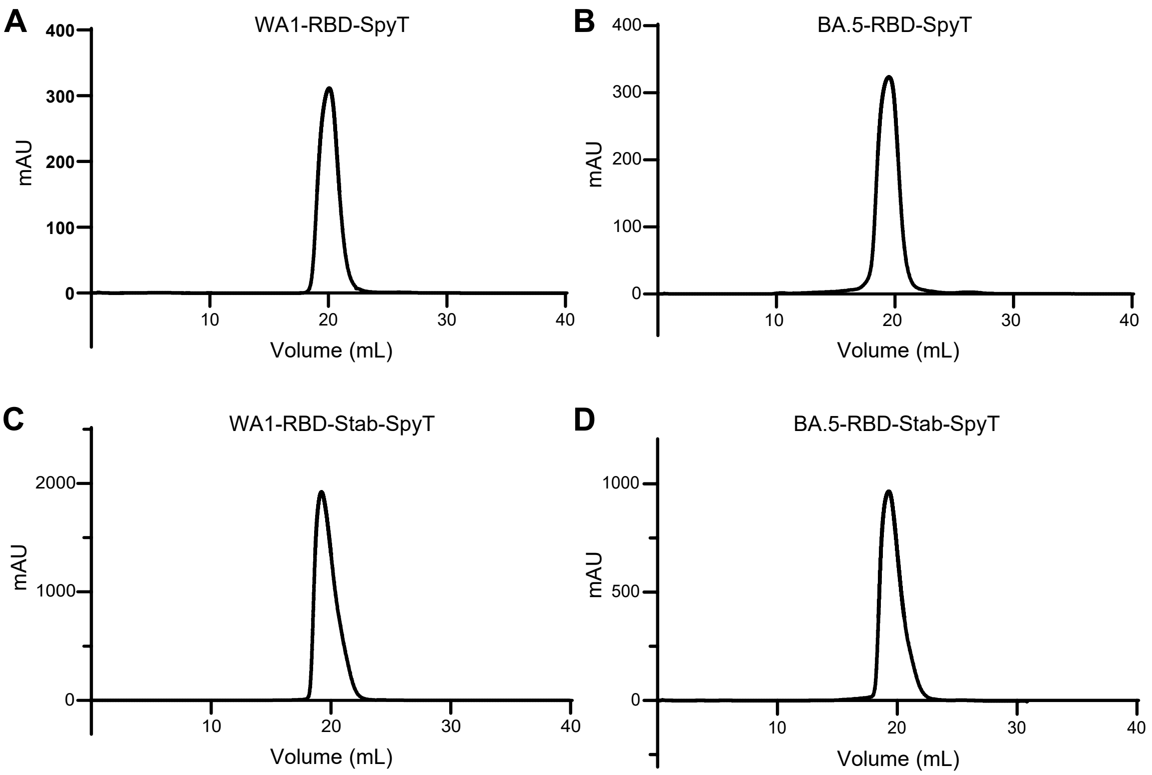

Figure S2. High purity RBD-SpyT proteins produced for nanoparticle preparation. SEC profiles of WA1-RBD-SpyT (A), BA.5-RBD-SpyT (B), WA1-RBD-Stab-SpyT (C), and BA.5-RBD-Stab-SpyT (D).

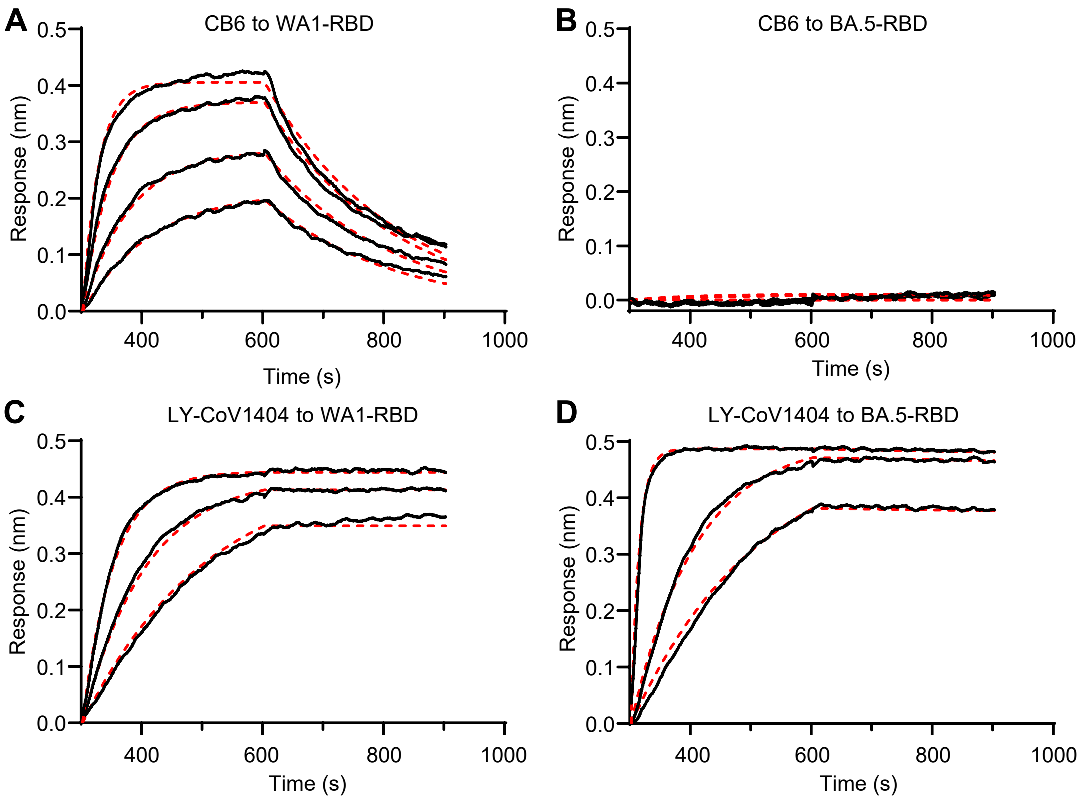

Figure S3. Antigenic analysis of different RBD proteins. (A-D) BLI measurements are shown in black with fitting provided with dotted red lines. (A) Antigenic data with CB6 antibody dipped into WA1-RBD. (B) Antigenic data with CB6 antibody dipped into BA.5-RBD. (C) Antigenic data with LY-CoV1404 antibody dipped into WA1-RBD. (D) Antigenic data with LY-CoV1404 antibody dipped into BA.5-RBD.

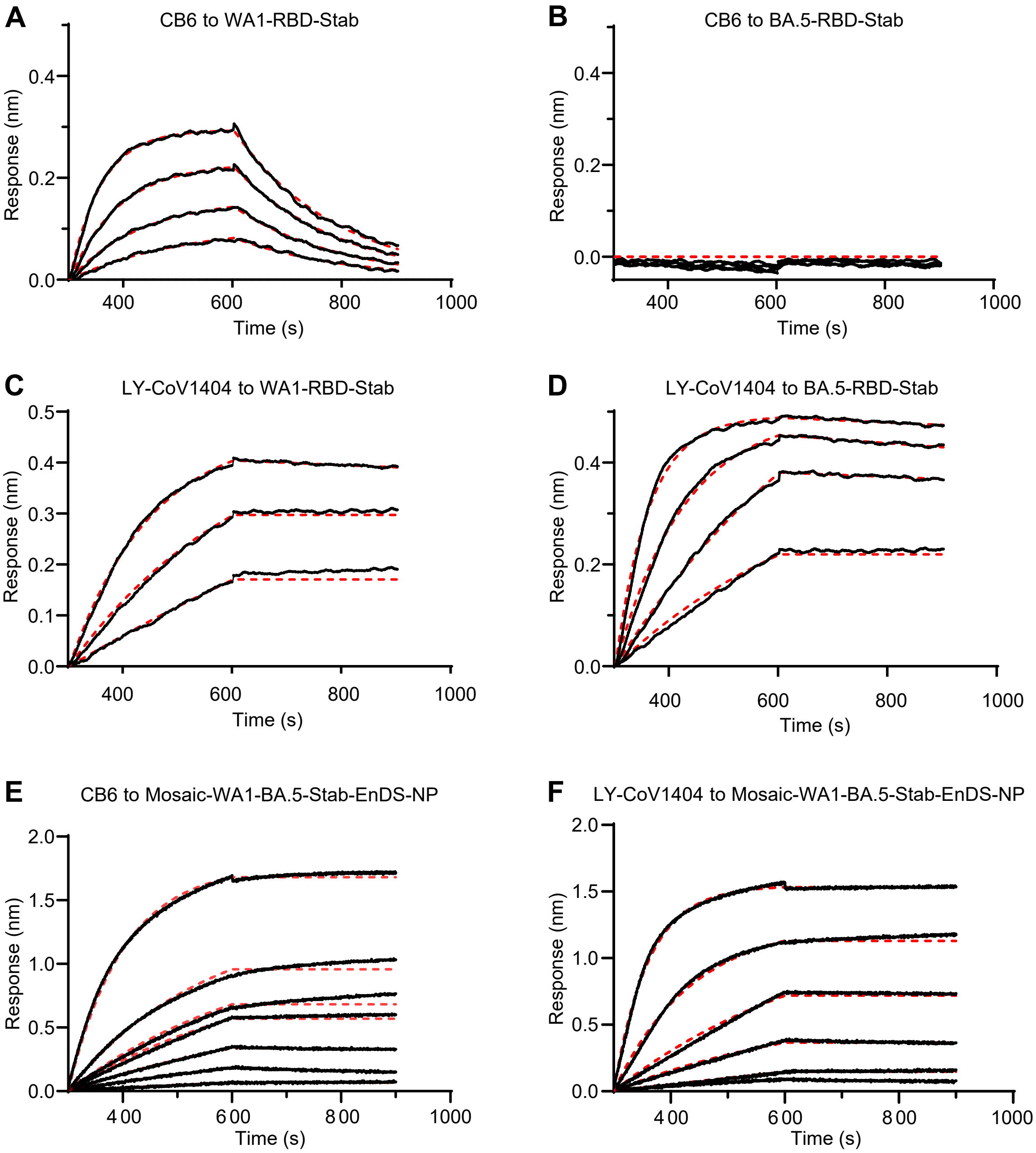

Figure S4. Antigenic analysis of different RBD proteins and Mosaic-WA1-BA.5-Stab-EnDS-NP. (A-F) BLI measurements are shown in black with fitting provided with dotted red lines. (A) Antigenic data with CB6 antibody dipped into WA1-RBD-Stab. (B) Antigenic data with CB6 antibody dipped into BA.5-RBD-Stab. (C) Antigenic data with LY-CoV1404 antibody dipped into WA1-RBD-Stab. (D) Antigenic data with LY-CoV1404 antibody dipped into BA.5-RBD-Stab. (E) Antigenic data with CB6 antibody dipped into Mosaic-WA1-BA.5-Stab-EnDS-NP. (F) Antigenic data with LY-CoV1404 antibody dipped into Mosaic-WA1-BA.5-Stab-EnDS-NP.

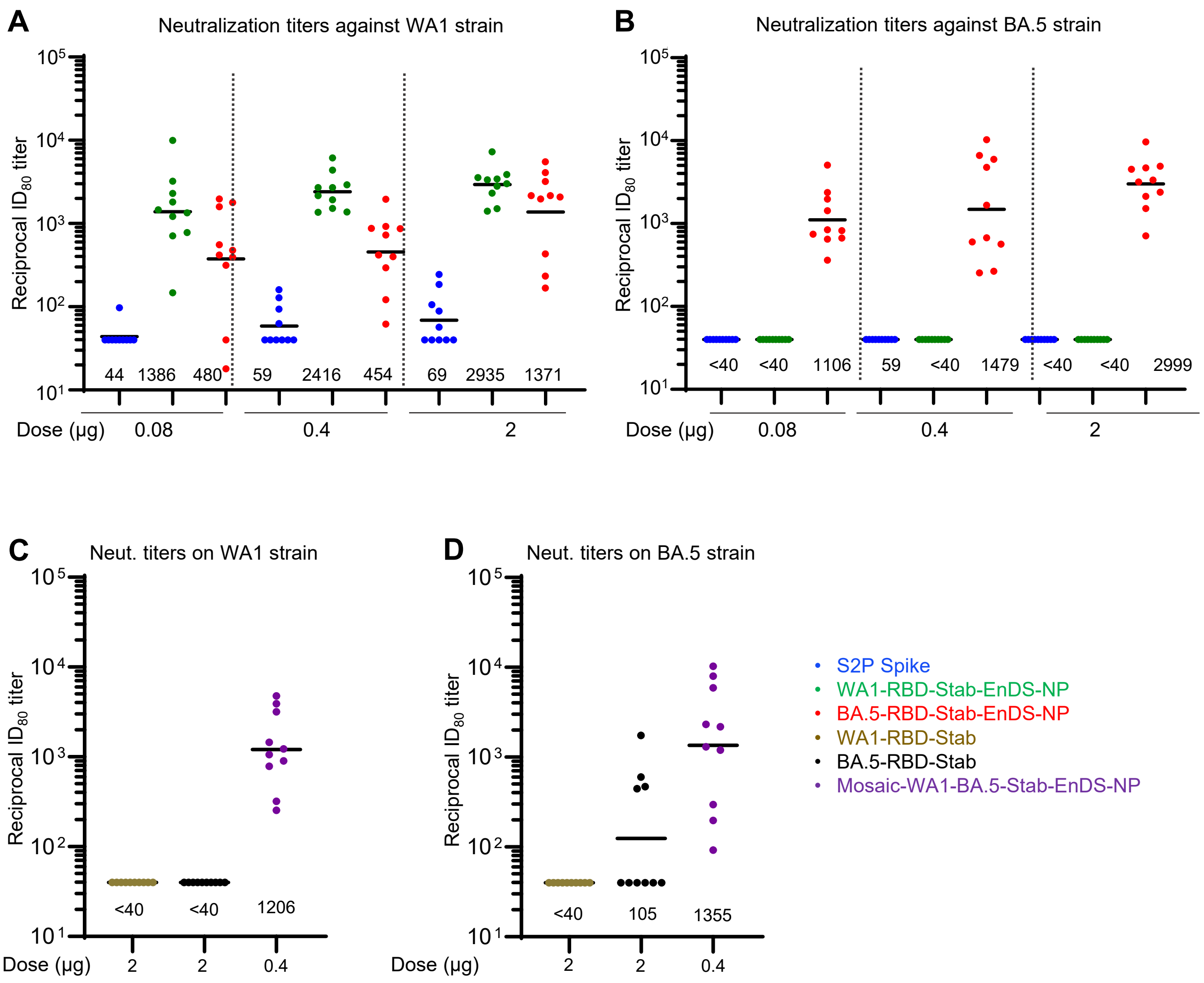

Figure S5. Immunization of stabilized RBD-EnDS nanoparticles elicited strong anti-SARS-CoV-2 pseudovirus neutralizing responses in mice. (A) Anti-SARS-CoV-2 WA1 pseudovirus neutralization ID80 titers from different immunogens. (B) Anti-SARS-CoV-2 BA.5 pseudovirus neutralization ID80 titers from different immunogens. (C) Anti-SARS-CoV-2 WA1 pseudovirus neutralization ID80 titers from different immunogens. (D) Anti-SARS-CoV-2 BA.5 pseudovirus neutralization ID80 titers from different immunogens.

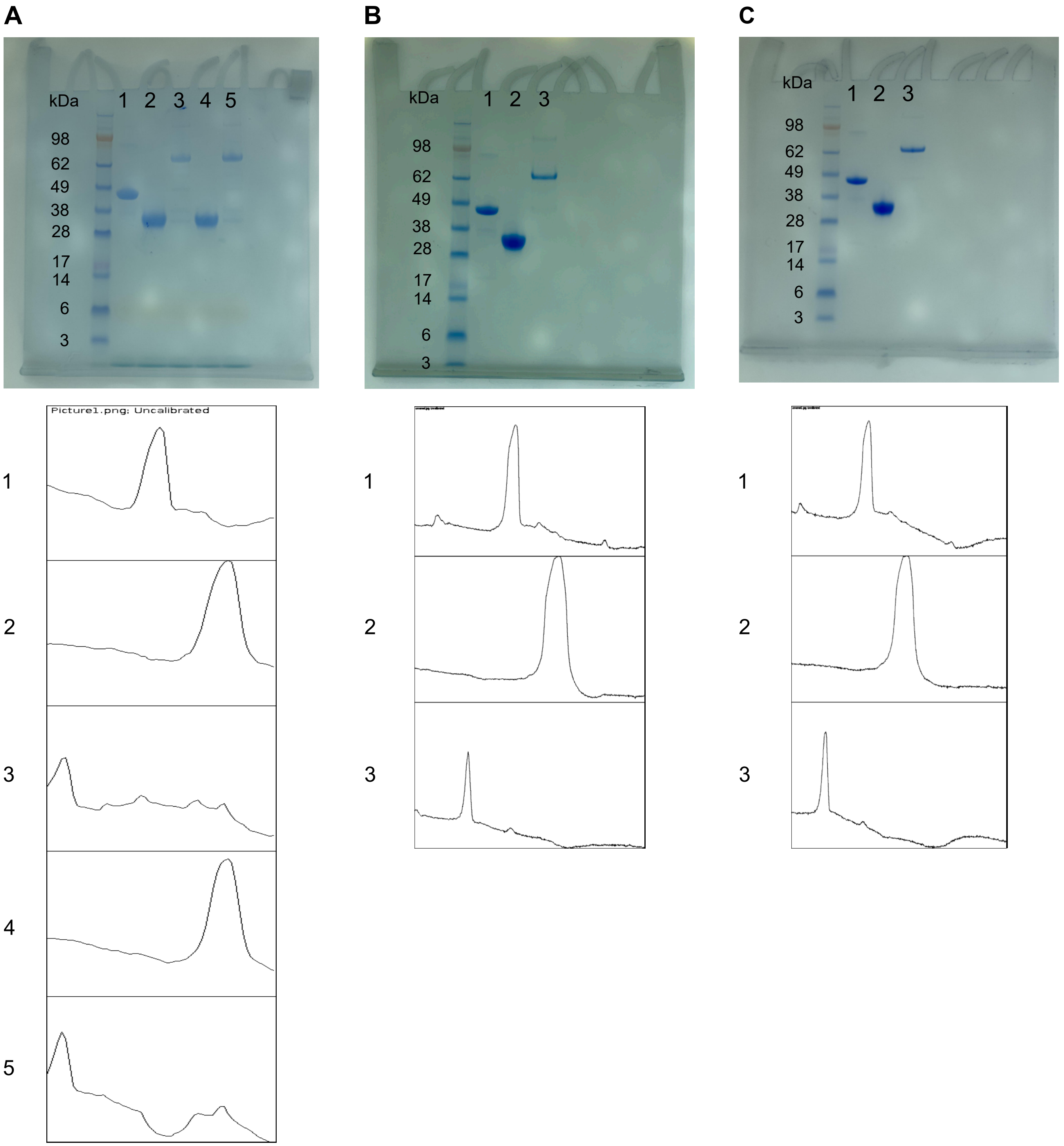

Figure S6. Uncropped SDS-PAGE images and densitometry readings of each lane. (A) Uncropped image of Figure 1C and densitometry readings of each lane. (B) Uncropped image of Figure 3E and densitometry readings of each lane. (C) Uncropped image of Figure 3F and densitometry readings of each lane. Molecular weight markers are show to the left of each gel.
